# Racial disparities in the association between adverse childhood experiences and history of self-reported depression among cancer survivors

**DOI:** 10.1007/s11764-025-01965-4

**Published:** 2026-01-16

**Authors:** Vincent J. Colucciello, Oluwole A. Babatunde, Katherine Gonzalez, Ngozi Adaralegbe, Melanie S. Jefferson, Swann A. Adams, Chanita Hughes Halbert, Frank Clark, Anusuiya Nagar, Nosayaba Osazuwa-Peters, Eric Adjei Boakye

**Affiliations:** 1 University of South Carolina School of Medicine Greenville, Greenville, SC USA; 2 Prisma Health, Greer, SC USA; 3 California University of Science and Medicine, San Bernardino, CA USA; 4 Department of Psychiatry and Behavioral Sciences, McGovern Medical School, Houston, TX USA; 5 Department of Psychiatry, Medical University of South Carolina, Charleston, SC USA; 6 Biobehavioral Health and Nursing Science Department, College of Nursing, University of South Carolina Columbia, Columbia, SC USA; 7 Department of Epidemiology & Biostatistics, University of South Carolina Columbia, Columbia, SC USA; 8 Department of Population & Public Health Sciences, Keck School of Medicine of University of Southern California, Los Angeles, CA USA; 9 USC Norris Comprehensive Cancer Center, Los Angeles, CA USA; 10 Department of Head and Neck Surgery & Communication Sciences, Duke University School of Medicine, Durham, NC USA; 11 Duke Cancer Institute, Durham, NC USA; 12 Department of Public Health Sciences, Henry Ford Health System, One Ford Place, Detroit, MI 48202 USA; 13 Department of Otolaryngology – Head and Neck Surgery, Henry Ford Health System, Detroit, MI USA; 14 Henry Ford Health + Michigan State University Health Sciences, Detroit, MI USA; 15 Department of Epidemiology and Biostatistics, Michigan State University College of Human Medicine, East Lansing, MI USA

**Keywords:** Adverse childhood experiences, Depression, Cancer survivors, Racial disparities

## Abstract

**Objective:**

This study aimed to assess the association between the number of adverse childhood experiences (ACEs) and the history of depression among cancer survivors by race.

**Methods:**

This study was a cross-sectional analysis of the 2020 Behavioral Risk Factor Surveillance System among 22,292 cancer survivors. The number of ACE experienced (zero, one, two-three, ≥ four) included questions assessing exposure to physical, emotional, and sexual household member substance misuse, incarceration, mental illness, parental divorce, or witnessing intimate partner violence before age 18 years. The outcome is self-reported history of depression (yes/no). Three weighted multivariable logistic regression models were used to examine the association between ACE and depression for each race group: non-Hispanic White (NHW), non-Hispanic Black (NHB), and Hispanics.

**Results:**

In this sample of cancer survivors, 41%, 22%, 21%, and 16% reported having experienced zero, one, two-three, and ≥ 4 ACEs, respectively. In the adjusted models, among NHW survivors, those who experienced ≥ 4 ACEs (aOR 4.20; 95% CI 3.32–5.32) had higher odds of depression diagnosis compared with those with zero ACEs. Among NHB survivors, those who experienced ≥ 4 ACEs had higher odds of depression diagnosis (aOR 2.98; 95% CI 1.45–6.14) compared with those with zero ACEs. Similarly, among Hispanic survivors, those who experienced ≥ 4 ACEs had higher odds of depression diagnosis (aOR 10.74; 95% CI 5.01–23.00) compared with those with zero ACEs.

**Conclusion:**

Individuals with ACEs may constitute a key group for targeted prevention efforts to mitigate the risk of depression in cancer survivors.

**Implications for Cancer Survivors:**

Development of personalized and targeted screening methods for patients diagnosed with cancer who have experienced adverse childhood experiences and depression is critical to improving their long-term well-being.

## Introduction

For more than a decade, researchers have investigated the impacts of adverse childhood experiences (ACEs) and their significance on public health and global health burden surveillance [1]. ACEs are defined as emotional, physical, or sexual abuse; neglect; substance misuse; and other traumatic events that occur to a child before the age of 18 [2]. Annually, nearly 600,000 documented cases of child abuse and neglect are reported [3, 4]. Additionally, 60% of American adults report experiencing at least one ACE, with 17% reporting four or more [3, 4]. ACEs have been linked to an increased risk of morbidity and mortality, contributing to conditions such as coronary ischemic heart disease, cancer, chronic lung disease, skeletal fractures, disability, and liver disease during adulthood [2, 5]. Additionally, ACEs are associated with mental health concerns, including depression, illicit drug use, and alcohol consumption [6–8].

Certain minority groups face a significantly higher risk of experiencing ACEs, which may be partly driven by discrimination [9, 10]. This discrimination can lead to economic disadvantages, which in turn increase the likelihood of encountering ACEs [11]. Minority communities also experience lower quality healthcare and inferior access to mental health resources compared to their white counterparts [12]. ACEs also vary in prevalence across different racial groups [13]. Among non-Hispanic Black children, 61% have experienced at least one ACE, compared to 51% of Hispanic children [13]. The prevalence is 40% for non-Hispanic White children and significantly lower at 23% for non-Hispanic Asian children [13]. Given that minority groups often face more severe and prolonged mental health challenges, it is crucial to identify risk factors to proactively address and mitigate their effects [14, 15].

Cancer survivors are at higher risk of developing mental health conditions, specifically depression, which is three times more likely to manifest in cancer survivors compared to the general population [16]. A robust association exists between the mortality rate in cancer survivors and the presence of minor or major depression, which can increase the mortality rate by 39% [17]. In the USA, 25% of cancer survivors experience depression [18], and there are racial disparities in how ACEs contribute to the development of depression in this population [14, 15]. In addition, existing literature has demonstrated increased risk of cancer in adults with certain types of ACEs such as physical and psychological abuse [19, 20]. While prior studies have explored the association between ACEs and depression within the general population, research focusing on cancer survivors stratified by racial differences is lacking. Our study addresses this gap by quantifying the risk of depression in cancer survivors across different racial groups based on the number of ACEs they experienced. Specifically, we evaluated the association between the number of ACEs experienced and a history of self-reported depression among cancer survivors from different racial/ethnic groups (non-Hispanic White, non-Hispanic Black, non-Hispanic Other, and Hispanic). This data may provide the empirical data necessary for developing race-specific, risk stratified screening methods for patients diagnosed with cancer who have experienced ACEs.

## Methods

### Data source and study sample

This was a cross-sectional analysis of data from the 2020 Behavioral Risk Factor Surveillance System (BRFSS). BRFSS is the largest continuously conducted health survey in the world, administered by state health departments with oversight from the Centers for Disease Control and Prevention (CDC), and reaches over 500,000 respondents. It is a state-based telephone survey of noninstitutionalized adults administered annually in the USA and associated territories. The BRFSS has three overall components: core modules (sets of questions consistently administered to all states and territories to establish national estimates), optional modules (CDC–developed questions that states can include in their BRFSS survey depending on their priorities), and state-added questions (state-customized items). Details of the survey methods have been published elsewhere [21]. This study utilized open-access de-identified data and was exempt from institutional review board approval. In 2020, 28 states (AL, AZ, CA, DC, FL, GA, HI, ID, IA, KS, KY, MD, MA, MS, MO, MT, NV, NJ, ND, OK, RI, SC, SD, TX, UT, VA, WI, WY) included state-added adverse childhood experience questions. For this study, we included 22,292 cancer survivors aged 18 and above. Respondents with missing ACE or depression data were excluded. Cancer survivors were identified with a yes response to the following question: “(Ever told) you had skin cancer or any other types of cancer?”

### Measures

#### Adverse childhood experiences

The primary exposure variable in this study was the number of ACEs. ACE was determined by responses to 11 questions that assessed exposure to eight types of adverse childhood experiences. These childhood experiences included three types of abuse and five types of household challenges before age 18. The three types of abuse considered included physical, emotional, and sexual. The five types of household challenges assessed included household member(s) of the respondent’s families with substance misuse, incarceration or mental illness, parental divorce, or witnessing intimate partner violence. Answers were coded as “one” if the ACE was present and “zero” if absent. The responses were summed to produce an ACE score, with cumulative scores ranging from zero to 11. We created categories of ACE scores as follows: zero; one; two-three; and ≥ four based on prior literature [22–25].

#### Depression

The outcome variable was a self-reported history of depression, assessed by response to the BRFSS question, “Has a doctor, nurse, or other health professionals ever told you that you had a depressive disorder (including depression, major depression, dysthymia, or minor depression)?” Response options were yes or no, and those who answered yes were classified as having a history of depression.

#### Race/ethnicity

Three race/ethnicity groups were selected a priori for this analysis: non-Hispanic White, non-Hispanic Black, Hispanic, and non-Hispanic Others.

#### Covariates

The following covariates, selected based on prior literature [26–32] were included in the study: age (18–39, 40–64, and ≥ 65), sex (female, male), marital status (married/partnered, divorced/separated/widowed, or never married), education level (college graduate, some college, or < high school (HS)/HS graduate), income level (≥ $50,000, $25,000 to 49,999, < $25,000, or missing), body mass index (BMI) (normal, overweight, or obese; BMI was defined as normal if 18.5 to < 25 kg/m^2^, overweight if 25.0 to < 30 kg/m^2^, and obese if 30.0 kg/m^2^ or higher), and smoking status (never, former, or current).

### Statistical analysis

Descriptive statistics were used to describe the characteristics of respondents overall and stratified by the number of ACEs experienced. Chi-square tests were used to compare the differences in covariates across the number of ACEs (zero, one, two–three, or ≥ four ACEs). Second, four weighted multivariable logistic regression models were used to examine the association between the number of ACEs and self-reported depression, one for each race/ethnicity, adjusting for age, sex, marital status, education level, income level, body mass index, smoking status, and health status. All analyses were weighted to account for the complex survey design of BRFSS and were performed using SAS version 9.4 (Cary, NC). *P*-values were two-sided, and *P* < 0.05 was considered statistically significant.

## Results

A total of 22,292 cancer survivors were included in the study, of whom 4338 (21.2%) reported a history of depression. Approximately 41%, 22%, 21%, and 16% reported having zero, one, two–three, or ≥ four ACEs, respectively. Table 1 summarizes the study sample’s characteristics, overall and stratified by number of ACEs. Overall, most respondents were 65 years or older (59.4%), female (56.0%), non-Hispanic whites (83.7%), married (61.4%), half were never smokers (49.9%), and there was equal distribution for BMI. In the bivariate analyses, respondents with depression were 18–39 years, female, non-Hispanic Blacks, never married, obese, current smokers, earned < $ 25 K, or reported fair/poor health.

**Table 1:** Sample characteristics, overall and stratified by history of depression, 2020 Behavioral Risk Factor Surveillance System (*n* = 22,292)

	**Overall (*n* = 22,292)**	**Depression**		*P***-value**
		**Yes (*n* = 4338)**	**No (*n* = 17,870)**	
**Depression diagnosis**				
Yes	4338 (21.2%)	–-	–-	
No	17,870 (78.8%)	–-	–-	
**Number of ACEs**				< 0.0001
Zero	9773 (41.1%)	1081 (11.7%)	8664 (88.3%)	
One	5088 (22.1%)	868 (18.4%)	4197 (81.6%)	
Two–Three	4375 (20.9%)	1059 (22.6%)	3296 (77.4%)	
> Four	3056 (15.9%)	1330 (47.8%)	1713 (52.2%)	
**Age at time of survey**				< 0.0001
18–39	675 (5.3%)	296 (45.3%)	374 (54.7%)	
40–64	6149 (32.3%)	1677 (28.1%)	4458 (71.9%)	
≥65	15,179 (59.4%)	2330 (15.2%)	12,787 (84.8%)	
**Sex**				< 0.0001
Female	12,690 (56.0%)	3038 (26.3%)	9612 (73.7%)	
Male	9602 (44.0%)	1300 (14.7%)	8258 (85.3%)	
**Race/ethnicity**				0.0021
Non-Hispanic White	19,427 (83.7%)	3681 (20.9%)	15,681 (79.1%)	
Non-Hispanic Black	914 (5.6%)	195 (30.1%)	714 (69.9%)	
Hispanic	576 (5.9%)	149 (15.7%)	424 (84.3%)	
Non-Hispanic Other	1375 (4.7%)	313 (24.1%)	1051 (75.9%)	
**Marital status**				< 0.0001
Married	12,759 (61.4%)	2130 (18.3%)	10,598 (81.7%)	
Divorced/separated/widowed	8004 (32.6%)	1825 (24.7%)	6137 (75.3%)	
Never married	1422 (6.0%)	370 (33.4%)	1048 (66.6)	
**Education level**				0.0005
College graduate	8992 (29.9%)	1530 (17.8%)	7437 (82.2%)	
Some college	6605 (33.3%)	1436 (22.0%)	5144 (78.0%)	
≤ High school graduate	6647 (36.8%)	1364 (23.3%)	5249 (76.7%)	
**Income level**				< 0.0001
≥ 50K	9326 (41.7%)	1444 (17.3%)	7862 (82.7%)	
25–50K	4858 (20.6%)	976 (23.1%)	3867 (76.9%)	
< 25K	4070 (20.2%)	1290 (33.1%)	2751 (66.9%)	
Refused or missing	4038 (17.6%)	628 (14.6%)	3390 (85.6%)	
**BMI category**				< 0.0001
Normal	6888 (31.3%)	1171 (19.0%)	5691 (81.0%)	
Overweight	7863 (36.1%)	1349 (18.1%)	6483 (81.9%)	
Obese	6383 (32.6%)	1581 (27.0%)	4782 (73.0%)	
**Smoking status**				< 0.0001
Never	11,797 (49.9%)	1906 (17.7%)	9849 (82.3%)	
Former	7986 (37.0%)	1542 (19.8%)	6418 (80.2%)	
Current	2364 (13.1%)	875 (39.0%)	1474 (61.0%)	
**Health status**				< 0.0001
Excellent/very good	9565 (40.8%)	1181 (12.3%)	8365 (87.7%)	
Good	7197 (32.2%)	1357 (22.1%)	5794 (77.9%)	
Fair/poor	5530 (27.0%)	1780 (33.7)	3711 (66.3%)	

*ACE*, adverse childhood experience

*P*-value based on the chi-square test

Table 2 summarizes multivariable logistic regression models for the association between the number of ACEs and history of depression stratified by race/ethnicity. Respondents who experienced ≥ four ACEs had a higher likelihood of reporting depression for all racial/ethnicity groups: non-Hispanic Whites (aOR = 4.20; 95% CI 3.32, 5.32), non-Hispanic Blacks (aOR = 2.98; 95% CI 1.45, 6.14), Hispanics (aOR = 10.74; 95% CI 5.01, 23.00), or non-Hispanic Others (aOR = 4.29; 95% CI 2.31, 7.98) compared to those who experienced zero ACEs. Respondents who experienced 2–3 ACEs had a higher likelihood of reporting depression for non-Hispanic Whites (aOR = 1.98; 95% CI 1.58, 2.48) compared to those who experienced zero ACEs. There was no statistically significance between experiencing 2–3 ACEs and depression for non-Hispanic Blacks, non-Hispanic Others, and Hispanics. Respondents who experienced one ACE had a higher likelihood of reporting depression for non-Hispanic Whites (aOR = 1.71; 95% CI 1.37, 2.14) compared to those who experienced zero ACEs, but no statistically significant difference was observed for non-Hispanic Blacks, non-Hispanic Others, and Hispanics.

**Table 2:** Four multivariable logistic models (one for each race/ethnicity) estimating associations between number of ACEs and history of depression, 2020 Behavioral Risk Factor Surveillance System (*n* = 22,292)

	Adjusted odds ratios (95% confidence interval)			
	Non-Hispanic White	Non-Hispanic Black	Hispanic	Non-Hispanic Others
**Number of ACEs**				
Zero	Ref	Ref	Ref	Ref
One	1.71 (1.37, 2.14)	0.94 (0.43, 2.04)	1.42 (0.53, 3.85)	1.44 (0.85, 2.46)
Two–Three	1.98 (1.58, 2.48)	0.98 (0.48, 2.03)	1.29 (0.48, 3.41)	1.02 (0.50, 2.08)
> Four	4.20 (3.32, 5.32)	2.98 (1.45, 6.14)	10.74 (5.01, 23.00)	4.29 (2.31, 7.98)
**Age at time of survey**				
≥65	Ref	Ref	Ref	Ref
18–39	3.59 (2.46, 5.22)	3.40 (0.99, 11.65)	0.67 (0.21, 2.11)	3.58 (1.65, 7.75)
40–64	1.51 (1.25, 1.82)	1.55 (0.91, 2.62)	2.76 (1.33, 5.73)	1.77 (1.06, 2.94)
**Sex**				
Female	2.17 (1.82, 2.58)	1.41 (0.66, 3.02)	3.44 (1.74, 6.77)	1.38 (0.91, 2.10)
Male	Ref	Ref	Ref	Ref
**Marital status**				
Married	Ref	Ref	Ref	Ref
Divorced/separated/widowed	1.18 (0.98, 1.41)	0.52 (0.24, 1.10)	2.04 (1.03, 4.13)	0.96 (0.61, 1.51)
Never married	1.49 (1.02, 2.18)	1.51 (0.49, 4.66)	1.66 (0.48, 5.77)	0.61 (0.22, 1.69)
**Education level**				
College graduate	Ref	Ref	Ref	Ref
Some college	0.87 (0.71, 1.05)	0.76 (0.35, 1.66)	2.20 (0.99, 4.92)	0.76 (0.42, 1.37)
≤ High school graduate	0.91 (0.72, 1.13)	0.69 (0.33, 1.44)	0.88 (0.34, 2.26)	0.88 (0.53, 1.45)
**Income level**				
≥ 50K	Ref	Ref	Ref	Ref
25–50K	1.20 (0.96, 1.49)	0.53 (0.26, 1.08)	0.58 (0.18, 1.91)	4.95 (2.56, 9.56)
< 25K	1.35 (1.07, 1.71)	1.13 (0.51, 2.51)	0.86 (0.28, 2.71)	2.32 (1.19, 4.52)
Refused/missing	0.73 (0.58, 0.93)	0.43 (0.17, 1.12)	0.82 (0.29, 2.38)	2.88 (1.38, 6.04)
**BMI category**				
Normal	Ref	Ref	Ref	Ref
Overweight	1.20 (1.00, 1.44)	0.77 (0.33, 1.82)	1.09 (0.46, 2.56)	1.47 (0.90, 2.38)
Obese	1.57 (1.29, 1.90)	0.59 (0.24, 1.46)	0.92 (0.36, 2.34)	3.95 (2.35, 6.64)
**Smoking status**				
Never	Ref	Ref	Ref	Ref
Former	1.11 (0.92, 1.34)	0.91 (0.51, 1.66)	0.70 (0.30, 1.61)	0.60 (0.36, 1.00)
Current	1.47 (1.15, 1.87)	1.63 (0.77, 3.49)	2.04 (0.93, 4.49)	1.99 (1.19, 3.33)
**Health status**				
Excellent/very good	Ref	Ref	Ref	Ref
Good	1.83 (1.49, 2.25)	4.33 (2.05, 9.14)	1.42 (0.58, 3.52)	1.29 (0.74, 2.26)
Fair/poor	2.99 (2.46, 3.65)	7.16 (3.12, 16.45)	1.78 (0.79, 4.15)	2.97 (1.79, 4.91)

*ACE *Adverse childhood experience

Non-Hispanic Whites (aOR = 1.51; 95% CI 1.25, 1.82), Hispanics (aOR = 2.75; 95% CI 1.33, 5.73), and non-Hispanic Others (aOR = 1.77; 95% CI 1.06, 2.94) aged 40–64 had a higher likelihood of reporting depression compared to their ≥ 65 counterparts. Additionally, non-Hispanic Whites (aOR = 3.59; 95% CI 2.46, 5.22) and non-Hispanic Others (aOR = 3.58; 95% CI 1.65, 7.75) aged 18–39 had a higher likelihood of reporting depression compared to their ≥ 65 counterparts. Non-Hispanic White females (aOR = 2.17; 95% CI 1.82, 2.58) and Hispanic females (aOR = 3.44; 95% CI 1.74, 6.77) had a higher likelihood of reporting depression compared to their male counterparts. Among non-Hispanic Whites who were never married (aOR = 1.49; 95% CI 1.02, 2.18) vs. married; earned < $ 25 K (aOR = 1.35; 95% CI 1.07, 1.71) vs. > $ 50 K; were obese (aOR = 1.57; 95% CI 1.29, 1.90) vs. normal; were current smokers (aOR = 1.47; 95% CI 1.15, 1.87) vs. never smokers; and had good (aOR = 1.83; 95% CI 1.49, 2.25) or fair/poor (aOR = 2.99; 95% CI 2.46, 3.65) vs. very good/excellent self-reported health status had a higher likelihood of reporting depression. Among non-Hispanic Blacks who had good (aOR = 4.33; 95% CI 2.05, 9.14) or fair/poor (aOR = 7.16; 95% CI 3.12, 14.4) vs. very good/excellent self-reported health status had a higher likelihood of reporting depression.

## Discussion

Recognition of causes of depression in patients diagnosed with cancer is important because depression in this population affects both the individual as well as the health care system. Individuals with depression have a higher risk of suicide [33], lower adherence to treatment [34], and increased mortality rates of up to 39% [17]. Depression in cancer survivors results in more frequent healthcare visits, increased emergency department visits, higher rates of hospital readmissions, longer hospital stays, and greater healthcare costs [35–37]. In this paper, we explored the impact of ACEs experienced during childhood on depression diagnosis in adult cancer survivors across different racial/ethnic groups. We found that overall, 21% of cancer survivors reported a history of depression, which is higher than the 18.5% of self-reported depression observed in the general US adult population [38]. However, it falls within the range of self-reported depression rates typically seen in the cancer population [39]. We found that irrespective of respondents’ race/ethnicity, experiencing a higher number of ACEs, specifically four or more, was associated with increased odds of reporting depression in adulthood among cancer survivors compared to those who did not experience any ACE. There were no significant differences in depression for non-Hispanic Blacks, non-Hispanic Others, and Hispanics with less than three ACEs. A previous meta-analysis of ACEs and risk of depression has found that this relationship is non-linear and there is a more pronounced increase in the risk of depression with subsequent numbers of ACES [40]. This in addition to healthy coping mechanisms and resilience may have led to no significant differences in depression at lower levels of ACEs among minoritized groups.

Understanding the full psychosocial history of cancer survivors allows providers to tailor care in a way that mitigates long-term mental health consequences and reduces costs. For instance, the National Comprehensive Cancer Network (NCCN) currently recommends distress screenings at every visit if possible, but that can be difficult, and there are no other recommended specific intervals [41]. Incorporating an ACE assessment into this workflow could identify patients who warrant prioritized attention regarding mood disorders. While our analysis established a significant association between a history of ACEs and depression among cancer survivors, it is important to acknowledge that this relationship is likely multifaceted. Several other factors that are often interrelated to mental health outcomes, such as socioeconomic factors, cancer-related stress, and access to quality care, could act as confounding variables. Future research should isolate these factors and their impact on ACEs and depression.

This study found that individuals from different racial/ethnic groups exhibited increased odds of depression with an increased number of ACEs. A previous longitudinal study of adolescents of African or European ancestry followed these participants into early adulthood (upper age of 42) and found no statistically significant association between a higher number of ACEs and depression [42]. The increased risk of developing depression in cancer survivors who experienced a higher number of ACEs may be due to biological. Previous studies have found that stressful events in childhood may lead to an overactivation of the hypothalamic-pituitary-adrenal (HPA) axis, which can persist into adulthood [43]. Changes in the HPA axis play a large role in the development of depression and are a key contributor to decreased cognitive function in depressed patients [44, 45]. Additionally, higher levels of stress have been associated with depression [46], and ACEs have a dose-dependent relationship with frequency of stress, severity of stress, and negative affect that persists in adulthood [47].

Age and sex were associated with depression among some respondents in this study. Non-Hispanic Whites ages 18–39 had a higher odds of reporting depression compared to their ≥ 65 counterparts; respondents of non-Hispanic Whites and Hispanics ages 40–64 had a higher odds of reporting depression compared to their ≥ 65 counterparts. This is not entirely surprising, given that the prevalence of depression is highest in young adulthood and lowest in individuals aged 65 and older [48]. This may be due to the way depression manifests in elderly patients. Older individuals are more likely to experience depression without the affective component, meaning they may have depression without the typical feelings of sadness [49]. Consequently, self-reported depression may be underreported in the older adult population [50]. Younger adults may be at a greater risk of depression due to poor coping strategies, which may elevate their risk of experiencing self-reported depression [51]. In this study, there was no difference in the rates of depression between different age groups of non-Hispanic Blacks. This may be due to younger non-Hispanic Blacks being more likely to utilize a variety of coping strategies such as psychological openness, informational support, religious coping, and seeking professional help compared to older non-Hispanic Black people [52], which may provide a protective role in this age group leading to no significant difference in depression between the different ages of non-Hispanic Black people.

In our study, both non-Hispanic white and Hispanic females had higher odds of reporting depression than males. The difference between men and women developing depression after facing ACEs is inconsistent in the current literature. Some studies have shown that there is no difference in the rate of depression between men and women later in life after experiencing ACES [53, 54]. However, other studies have found that females have higher rates of depression compared to males [55, 56]. In the general population, female sex hormones can increase the risk of depression due to higher levels of neuro-immune response [57]. While depression is diagnosed twice as frequently in females compared to males [58], males have a higher rate of undiagnosed depression [58, 59] and are less likely to seek medical help compared to women [60]. Additionally, a previous study has found that ACEs affect men and women differently later in life [61, 62]. Women more frequently develop psychological disorders, while men develop substance use disorders [62] which may be a confounding variable for the diagnosis of mood disorders [63]. However, one study has found that non-Hispanic Black women are more open to receiving professional help and are more psychologically open in terms of mental health disorders compared to men [52]. Additionally, non-Hispanic Black women have greater economic advantages and higher education compared to non-Hispanic Black men [64, 65], both of which may be protective factors for depression [66]. These protective factors in non-Hispanic Black women may have led to lower levels of depression in our study, which could cause no significant difference between the depression of non-Hispanic Black women compared to non-Hispanic Black men.

### Limitations and strengths

This study has limitations. First, BRFSS did not have information on the severity of cancer, and thus, we could not adjust for it in the analyses, which could influence findings on depression. Second, ACEs and depression were self-reported; relying on self-reported data introduces potential biases, such as misclassification or recall bias. This method leaves room for both overestimation or underestimation of ACE exposure or depression. Third, the depression variable used in this study is a single-item measure, although it has been used in several published studies. Moreover, the dataset does not contain information on the onset or severity of the depression among respondents or treatment received by respondents. Future research should use validated screening tools (e.g., Patient Health Questionnaire-9 (PHQ-9), the Beck Depression Inventory (BDI), and the Center for Epidemiologic Studies Depression Scale (CES-D)) to provide a more nuanced understanding of depression in this population. Fourth, the dataset does not contain information on the age at which ACEs occurred, which could have affected the findings. Future research should evaluate if ACEs experienced in childhood had different psychological impacts than experiences during adolescence. Fifth, our analysis was cross-sectional, meaning that while associations were drawn, we cannot establish temporal significance or causality for outcomes. Despite these limitations, one main strength is that our study is one of the first to explore the association between ACEs and self-reported depression among different racial/ethnic cancer survivors. Secondly, this study had a large sample size, which reduces the risk of a statistical difference being due to sampling error. Our findings fill gaps in existing literature and offer insights that could guide the development of targeted prevention interventions for populations who experienced ACEs, potentially reducing the risk of depression in adult cancer patients.

## Conclusion

An increased number of ACEs were associated with a higher likelihood of self-reporting depression diagnoses across all race/ethnicity groups in our study. Cancer survivors are deemed vulnerable in terms of health, given the adverse impact of cancer on both physical and mental well-being. Additional studies are warranted to explore potential social factors contributing to this heightened risk and to develop intervention strategies aimed at mitigating this risk.

## Data Availability

The data used in this study are publicly available and can be accessed from the BRFSS website at [https://www.cdc.gov/brfss/data_documentation/index.htm].

## References

[1] Anda RF, Butchart A, Felitti VJ, Brown DW. Building a framework for global surveillance of the public health implications of adverse childhood experiences. Am J Prev Med. 2010;39(1):93–8. 10.1016/j.amepre.2010.03.015 20547282

[2] Felitti VJ, Anda RF, Nordenberg D, et al. Relationship of childhood abuse and household dysfunction to many of the leading causes of death in adults: the adverse childhood experience study. Am J Prev Med. 1998;14(4):245–58. 10.1016/s0749-3797(98)00017-8 9635069

[3] U.S. Department of Health & Human Services, Administration for Children and Families, Administration on Children, Youth and Families, Children’s Bureau. (2023). Child Maltreatment 2021. Available from https://www.acf.hhs.gov/cb/data-research/child-maltreatment.

[4] Swedo EA, Aslam MV, Dahlberg LL, Niolon PH, Guinn AS, Simon TR, Mercy JA. Prevalence of adverse childhood experiences among U.S. adults - Behavioral Risk Factor Surveillance System, 2011–2020. MMWR Morb Mortal Wkly Rep. 2023 Jun 30;72(26):707–715 10.15585/mmwr.mm7226a2 PMC10328489 37384554

[5] Campbell JA, Walker RJ, Egede LE. Associations between adverse childhood experiences, high-risk behaviors, and morbidity in adulthood. Am J Prev Med. 2016;50(3):344–52. 10.1016/j.amepre.2015.07.022. PMC4762720 26474668

[6] Kim Y, Lee H, Park A. Patterns of adverse childhood experiences and depressive symptoms: self-esteem as a mediating mechanism. Soc Psychiatry Psychiatr Epidemiol. 2022;57(2):331–41. 10.1007/s00127-021-02129-2 PMC8243305 34191037

[7] Dube SR, Felitti VJ, Dong M, Chapman DP, Giles WH, Anda RF. Childhood abuse, neglect, and household dysfunction and the risk of illicit drug use: the adverse childhood experiences study. Pediatrics. 2003;111:564–72. 10.1542/peds.111.3.564. 12612237

[8] Crouch E, Radcliff E, Strompolis M, Wilson A. Adverse childhood experiences (ACEs) and alcohol abuse among South Carolina adults. Subst Use Misuse. 2018Jun 7;53(7):1212–20. 10.1080/10826084.2017.1400568 29185846

[9] Merrick MT, Ford DC, Ports KA, Guinn AS. Prevalence of adverse childhood experiences from the 2011-2014 behavioral risk factor surveillance system in 23 states. JAMA Pediatr. 2018;172(11):1038–44. 10.1001/jamapediatrics.2018.2537. PMC6248156 30242348

[10] Darity W Jr. Stratification economics: the role of intergroup inequality. Journal of Economics and Finance. 2005;29(2):144–53.

[11] Drake, Brett & Jonson-Reid, Melissa. (2014). Poverty and child maltreatment. 10.1007/978-94-007-7208-3_7.

[12] Samnaliev M, McGovern MP. Racial/ethnic disparities in mental health treatment in six Medicaid programs. J Health Care Poor Underserved. 2009;20:165–76. 10.1353/hpu.0.0125 19202255

[13] Sacks V, Murphey D. The prevalence of adverse childhood experiences, nationally, by state, and by race or ethnicity. https://www.childtrends.org/publications/prevalence-adverse-childhood-experiences-nationally-state-race-ethnicity. Accessed 1/05/2026.

[14] Williams DR, González HM, Neighbors H, et al. Prevalence and distribution of major depressive disorder in African Americans, Caribbean Blacks, and Non-Hispanic Whites: results from the National Survey of American Life. Arch Gen Psychiatry. 2007;64(3):305–15. 10.1001/archpsyc.64.3.305 17339519

[15] Babatunde OA, Gonzalez K, Osazuwa-Peters N, Adams SA, Hughes Halbert C, Clark F, et al. Adverse childhood events significantly impact depression and mental distress in adults with a history of cancer. Cancers (Basel). 2024. 10.3390/cancers16193290. PMC11476032 39409912

[16] Linden W, Vodermaier A, Mackenzie R, Greig D. Anxiety and depression after cancer diagnosis: prevalence rates by cancer type, gender, and age. J Affect Disord. 2012;141:343–51. 10.1016/j.jad.2012.03.025. 22727334

[17] Satin JR, Linden W, Phillips MJ. <article-title update="added">Depression as a predictor of disease progression and mortality in cancer patients: a meta‐analysis. Cancer. 2009;115:5349–61. 10.1002/cncr.24561 19753617

[18] Hartung TJ, Brähler E, Faller H, et al. The risk of being depressed is significantly higher in cancer patients than in the general population: prevalence and severity of depressive symptoms across major cancer types. Eur J Cancer. 2017;72:46–53. 10.1016/j.ejca.2016.11.017 28024266

[19] Holman DM, Ports KA, Buchanan ND, Hawkins NA, Merrick MT, Metzler M, et al. The association between adverse childhood experiences and risk of cancer in adulthood: a systematic review of the literature. Pediatrics. 2016;138(Supplement_1):S81–91. 10.1542/peds.2015-4268L PMC5892430 27940981

[20] Ports KA, Holman DM, Guinn AS, Pampati S, Dyer KE, Merrick MT, et al. Adverse childhood experiences and the presence of cancer risk factors in adulthood: a scoping review of the literature from 2005 to 2015. J Pediatr Nurs. 2019;44:81–96. 10.1016/j.pedn.2018.10.009 PMC6355255 30683285

[21] Mokdad AH. The behavioral risk factors surveillance system: past, present, and future. Annu Rev Public Health. 2009;30:43–54. 10.1146/annurev.publhealth.031308.100226. 19705555

[22] Ford DC, Merrick MT, Parks SE, et al. Examination of the factorial structure of adverse childhood experiences and recommendations for three subscale scores. Psychol Violence. 2014;4(4):432–44. 10.1037/a0037723. PMC4587306 26430532

[23] ​Merrick MT, Ford DC, Ports KA, Guinn AS, Chen J, Klevens J, et al. Vital signs: estimated proportion of adult health problems attributable to adverse childhood experiences and implications for prevention - 25 States, 2015-2017. MMWR Morb Mortal Wkly Rep. 2019;68(44):999–1005. 10.15585/mmwr.mm6844e1 PMC6837472 31697656

[24] Sonu S, Post S, Feinglass J. Adverse childhood experiences and the onset of chronic disease in young adulthood. Prev Med. 2019;123:163–70. 10.1016/j.ypmed.2019.03.032. 30904602

[25] Babatunde OA, Ramkumar SP, Nguyen SA, Okereke OI, Clark FA, Nagar A, et al. Association between number of adverse childhood experiences and depression among older adults is moderated by race. Prev Med. 2024;181:107921. 10.1016/j.ypmed.2024.107921 38423302

[26] Ege MA, Messias E, Thapa PB, Krain LP. Adverse childhood experiences and geriatric depression: results from the 2010 BRFSS. Am J Geriatr Psychiatry. 2015;23(1):110–4. 10.1016/j.jagp.2014.08.014. PMC4267899 25306195

[27] Zheng X, Cui Y, Xue Y, et al. Adverse childhood experiences in depression and the mediating role of multimorbidity in mid-late life: a nationwide longitudinal study. J Affect Disord. 2022;301:217–24. 10.1016/j.jad.2022.01.040. 35031336

[28] VanBronkhorst SB, Abraham E, Dambreville R, Ramos-Olazagasti MA, Wall M, Saunders DC, et al. Sociocultural risk and resilience in the context of adverse childhood experiences. JAMA Psychiatry. 2024;81(4):406–13. 10.1001/jamapsychiatry.2023.4900. PMC10753442 38150238

[29] Whitaker RC, Dearth-Wesley T, Herman AN, et al. The interaction of adverse childhood experiences and gender as risk factors for depression and anxiety disorders in US adults: a cross-sectional study. BMC Public Health. 2021. 10.1186/s12889-021-12058-z. PMC8590371 34772386

[30] Sheffler J, Meyer C, Puga F. Multi-sample assessment of stress reactivity as a mediator between childhood adversity and mid- to late-life outcomes. Aging Ment Health. 2022;26(6):1207–16. 10.1080/13607863.2021.1910787. 33860705

[31] Nishimi K, Choi KW, Davis KA, Powers A, Bradley B, Dunn EC. Features of childhood maltreatment and resilience capacity in adulthood: results from a large community-based sample. J Trauma Stress. 2020;33(5):665–76. 10.1002/jts.22543. PMC7828462 32537815

[32] Deng K, Lacey RE. Adverse childhood experiences, child poverty, and adiposity trajectories from childhood to adolescence: evidence from the Millennium Cohort Study. Int J Obes. 2022;46(10):1792–800. 10.1038/s41366-022-01185-1. PMC9492536 35840773

[33] Yousaf U, et al. Suicides among Danish Cancer Patients 1971–1999. Br J Cancer. 2005;92(6):995–1000. 10.1038/sj.bjc.6602424. PMC2361949 15756279

[34] DiMatteo M. Robin, et al. Depression is a risk factor for noncompliance with medical treatment. Arch Intern Med. 2000;160(14):2101. 10.1001/archinte.160.14.2101. 10904452

[35] Prieto JM, Blanch J, Atala J, et al. Psychiatric morbidity and impact on hospital length of stay among hematologic cancer patients receiving stem-cell transplantation. J Clin Oncol. 2002;20:1907–17. 10.1200/JCO.2002.07.101 11919251

[36] Mausbach BT, Irwin SA. Depression and healthcare service utilization in patients with cancer. Psycho-Oncology. 2017;26:1133–9. 10.1002/pon.4133. PMC5074898 27102731

[37] Pan X, Sambamoorthi U. Health care expenditures associated with depression in adults with cancer. J Community Support Oncol. 2015;13:240. 10.12788/jcso.0150. PMC4576451 26270540

[38] Lee B, Wang Y, Carlson SA, Greenlund KJ, Lu H, Liu Y, et al. National, state-level, and county-level prevalence estimates of adults aged ≥18 years self-reporting a lifetime diagnosis of depression — United States, 2020. MMWR Morb Mortal Wkly Rep. 2023;72(24):644–50. 10.15585/mmwr.mm7224a1. PMC10328468 37318995

[39] Krebber AMH, et al. “Prevalence of depression in cancer patients: a meta-analysis of diagnostic interviews and self-report instruments.” Psycho-Oncology. 2013;23(2):121–30. 10.1002/pon.3409. PMC4282549 24105788

[40] Tan M, Mao P. Type and dose-response effect of adverse childhood experiences in predicting depression: A systematic review and meta-analysis. Child Abuse Negl. 2023;139:106091. 10.1016/j.chiabu.2023.106091. 36787671

[41] Hurst K. U.S. women are outpacing men in college completion, including in every major racial and ethnic group. https://www.pewresearch.org/short-reads/2024/11/18/us-women-are-outpacing-men-in-college-completion-includingin-every-major-racial-and-ethnic-group/. Accessed 01/05/2026.

[42] Youssef NA, Belew D, Hao G, Wang X, Treiber FA, Stefanek M, et al. Racial/ethnic differences in the association of childhood adversities with depression and the role of resilience. J Affect Disord. 2017;208:577–81. 10.1016/j.jad.2016.10.024. PMC5154886 27806896

[43] Heim C, Newport DJ, Heit S, Graham YP, Wilcox M, Bonsall R, et al. Pituitary–adrenal and autonomic responses to stress in women after sexual and physical abuse in childhood. JAMA. 2000;284:592–7. 10.1001/jama.284.5.592 10918705

[44] Mikulska J, Juszczyk G, Gawrońska-Grzywacz M, Herbet M. HPA axis in the pathomechanism of depression and schizophrenia: new therapeutic strategies based on its participation. Brain Sci. 2021;11(10):1298. 10.3390/brainsci11101298. PMC8533829 34679364

[45] Holsboer F. The corticosteroid receptor hypothesis of depression. Neuropsychopharmacology. 2000;23:477–501. 10.1016/S0893-133X(00)00159-7. 11027914

[46] Fuchs E, Flügge G. Cellular consequences of stress and depression. Dialogues Clin Neurosci. 2004;6(2):171–83. 10.31887/dcns.2004.6.2/efuchs. PMC3181796 22033809

[47] Mosley-Johnson E, Campbell JA, Garacci E, Walker RJ, Egede LE. Stress that endures: influence of adverse childhood experiences on daily life stress and physical health in adulthood. J Affect Disord. 2021;284:38–43. 10.1016/j.jad.2021.02.018. PMC8040325 33582431

[48] Lee B. National, state-level, and county-level prevalence estimates of adults aged ≥18 years self-reporting a lifetime diagnosis of depression — United States, 2020. MMWR Morb Mortal Wkly Rep. 2023. 10.15585/mmwr.mm7224a1. PMC10328468 37318995

[49] Birrer RB, Vemuri SP. Depression in later life: a diagnostic and therapeutic challenge. Am Fam Physician. 2004;69(10):2375–82. 15168957

[50] Alexopoulos GS. Depression in the elderly. Lancet. 2005;365:1961–70. 10.1016/S0140-6736(05)66665-2 15936426

[51] Aldwin C, Choun S, Spiro A. The coping, appraisal, and resilience in aging (CARA) model: longitudinal findings from the Normative Aging Study. Psychol Aging. 2024;39(8):884–96. 10.1037/pag0000863. PMC12021528 39666488

[52] Ward EC, Wiltshire JC, Detry MA, Brown RL. African American men and women’s attitude toward mental illness, perceptions of stigma, and preferred coping behaviors. Nurs Res. 2013;62(3):185–94. 10.1097/NNR.0b013e31827bf533. PMC4279858 23328705

[53] Arnow BA, Blasey CM, Hunkeler EM, Lee J, Hayward C. Does gender moderate the relationship between childhood maltreatment and adult depression? Child Maltreat. 2011;16(3):175–83. 10.1177/1077559511412067 21727161

[54] Sunley AK, Lok A, White MJ, Snijder MB, van Zuiden M, Zantvoord JB, et al. Ethnic and sex differences in the association of child maltreatment and depressed mood. The Helius Study. Child Abuse Negl. 2020;99:104239. 10.1016/j.chiabu.2019.104239. 31731139

[55] Assini-Meytin LC, Fix RL, Green KM, Nair R, Letourneau EJ. Adverse childhood experiences, mental health, and risk behaviors in adulthood: exploring sex, racial, and ethnic group differences in a nationally representative sample. J Child Adolesc Trauma. 2021;15(3):833–45. 10.1007/s40653-021-00424-3. PMC9360354 35958728

[56] Whitaker RC, Dearth-Wesley T, Herman AN, Block AE, Holderness MH, Waring NA, et al. The interaction of adverse childhood experiences and gender as risk factors for depression and anxiety disorders in US adults: a cross-sectional study. BMC Public Health. 2021;21:1–12. 10.1186/s12889-021-12058-z PMC8590371 34772386

[57] Goldstein JM, Holsen L, Handa R, Tobet S. Fetal hormonal programming of sex differences in depression: linking women’s mental health with sex differences in the brain across the lifespan. Front Neurosci. 2014;8:247. 10.3389/fnins.2014.00247. PMC4157606 25249929

[58] Parker G, Brotchie H. Gender differences in depression. Int Rev Psychiatry. 2010;22:429–36. 10.3109/09540261.2010.492391. 21047157

[59] Angst J, Gamma A, Gastpar M, Lépine JP, Mendlewicz J, Tylee A. Gender differences in depression: epidemiological findings from the European DEPRES I and II studies. Depression Research in European Society Study. 2002;5:201–9. 10.1007/s00406-002-0381-6 12451460

[60] Potts MK, Burnam MA, Wells KB. Gender differences in depression detection: a comparison of clinician diagnosis and standardized assessment. Psychol Assess. 1991;3:609–15.

[61] Addis ME, Mahalik JR. Men, masculinity, and the contexts of help seeking. Am Psychol. 2003;58:5–14. 10.1037/0003-066X.58.1.5. 12674814

[62] Almuneef Maha, et al. Gender-based disparities in the impact of adverse childhood experiences on adult health: findings from a national study in the Kingdom of Saudi Arabia. Int J Equity Health. 2017. 10.1186/s12939-017-0588-9. PMC5450265 28558774

[63] Quello S, Brady K, Sonne S. Mood disorders and substance use disorder: a complex comorbidity. Science & Practice Perspectives. 2005;3(1):13–21. 10.1151/spp053113. PMC2851027 18552741

[64] Young Black women are significantly outpacing black men in educational attainment. The Journal of Blacks in Higher Education. (2024, December 4). https://jbhe.com/2024/12/young-black-women-are-significantly-outpacing-black-men-in-educational-attainment/

[65] Bowie L. The economic status of Black women in America, 2011. https://thebrockreport.net/theeconomic-status-of-black-women-in-america/. Accessed 01/05/2026

[66] Ridley, M., Rao G, Schilbach F, Patel V. Poverty, depression, and anxiety: Causal evidence and mechanisms. Science. 2020;370(6522). https://pubmed.ncbi.nlm.nih.gov/33303583/10.1126/science.aay0214 33303583

